# Evaluation and management of lead exposure

**DOI:** 10.1186/s40557-015-0085-9

**Published:** 2015-12-15

**Authors:** Hwan-Cheol Kim, Tae-Won Jang, Hong-Jae Chae, Won-Jun Choi, Mi-Na Ha, Byeong-Jin Ye, Byoung-Gwon Kim, Man-Joong Jeon, Se-Yeong Kim, Young-Seoub Hong

**Affiliations:** Department of Occupational & Environmental Medicine, Inha University Hospital, 27, Inhang-Ro, Jung-Gu, Incheon, Republic of Korea; Department of Occupational and Environmental Medicine, College of Medicine, The Catholic University, 222, Banpo-Daero, Seocho-gu, Seoul South Korea; Department of Occupational and Environmental Medicine, KS Hospital, 220, Wangbeodeul-ro, Gwangsan-gu, Gwangju, South Korea; Department of Occupational & Environmental Medicine, Gachon University Gil Medical Center, 21, Namdong-daero 774beon-gil, Namdong-gu, Incheon, South Korea; Department of Preventive Medicine, College of Medicine, Dankook University, 119, Dandae-ro, Dongnam-gu, Cheonan-si, Chungcheongnam-do South Korea; Department of Occupational and Environmental Medicine, Dong-A University Hospital, 26, Daesingongwon-ro, Seo-gu, Busan, South Korea; Department of Preventive Medicine, Collage of Medicine, Dong-A University, 32, Daesingongwon-ro, Seo-gu, Busan, South Korea; Department of Occupational & Environmental Medicine, Collage of Medicine, Young-Nam University, Daegu, Republic of Korea; Department of Occupational & Environmental Medicine & Institute of Environmental and Occupational Medicine, Pusan Paik Hospital, InJe University, 75, Bokji-ro, Busanjin-gu, Busan, South Korea

**Keywords:** Antioxidants, Chelation, Environmental, Exposure, Lead

## Abstract

Lead, which is widely used in industry, is a common element found in low concentrations in the Earth’s crust. Implementations to reduce environmental lead concentrations have resulted in a considerable reduction of lead levels in the environment (air) and a sustained reduction in the blood lead levels of the average citizen. However, people are still being exposed to lead through a variety of routes in everyday commodities.

Lead causes health problems such as toxicity of the liver, kidneys, hematopoietic system, and nervous system. Having a carcinogenic risk as well, the IARC classifies inorganic lead compounds as probably carcinogenic to humans (Group 2A). Occupational lead poisonings have decreased due to the efforts to reduce the lead concentrations in the working environment. In contrast, health hazards associated with long-term environmental exposure to low concentrations of lead have been reported steadily. In particular, chronic exposure to low concentrations of lead has been reported to induce cognitive behavioral disturbances in children.

It is almost impossible to remove lead completely from the human body, and it is not easy to treat health hazards due to lead exposure. Therefore, reduction and prevention of lead exposure are very important. We reviewed the toxicity and health hazards, monitoring and evaluation, and management of lead exposure.

## Background

Lead is a common element found in low concentrations in the Earth’s crust. It is widely used in industry, particularly in products such as construction materials, paint, batteries, and piping. Lead causes health problems such as toxicity of the liver, kidneys, hematopoietic system, and nervous system. Having a carcinogenic risk as well, the IARC classifies inorganic lead compounds as probably carcinogenic to humans (Group 2A) [1]. Children, in particular, are at risk of developing cognitive behavioral function problems, even when exposed to low concentrations [2].

During the 1970’s and 80’s, concern over lead related health problems led to the implementation of policies, such as the introduction of unleaded gasoline and a ban on using lead-based paint. This has resulted in a considerable reduction of lead levels in the environment (air) and a sustained reduction in the blood lead levels of the average citizen [3]. However, people are still being exposed to lead through a variety of routes, such as secondhand exposure to pollution in the workplace, everyday household products, children’s toys, and in everyday commodities [4].

There has been much research conducted on the occupational and environmental exposure to lead, as well as its health impact, treatment, and management. Recently, research has been conducted on the management of low-level lead exposure. This study sought to examine and organize the results of recent research on the diagnosis and health impact of lead, as well as its treatment and management.

### Mechanism of toxicity

Toxicity is known to occur through lead-induced oxidative stress [5]. The mechanism of lead-induced oxidative stress has been described as the depletion of antioxidative reserves and the increased generation of reactive oxygen species (ROS), such as hydroperoxides [6]. Ninety percent of glutathione (GSH) in the cell exists in reduced form and 10 % in oxidative form, and it typically acts as an antioxidant defense mechanism. Reacting to ROS, GSH stabilizes ROS, and after being converted (oxidizing) to glutathione disulfide (GSSG), it is reduced back to GSH by glutathione reductase (GR). Lead inactivates GSH by binding to GSH’s sulfhydryl group, which causes GSH replenishment to become inefficient, thereby increasing oxidative stress [7]. In addition, lead further reduces glutathione levels by blocking the activity of enzymes such as δ-aminolevulinic acid dehydratase (ALAD), glutathione reductase (GR), glutathione peroxidase, and glutathione-S-transferase [8]. Lead destabilizes the cellular membrane through lipid peroxidation, which can lead to hemolytic anemia [9]. The collective action of various such mechanisms makes the cell vulnerable to oxidative stress, causing cell death.

Toxicity also arises from lead replacing divalent cations, which are necessary for cellular activity. The main mechanism causing neurological abnormalities is the ionic mechanism that substitutes lead for calcium ions, allowing it to pass through the blood-brain barrier [10]. By penetrating the blood-brain barrier, lead accumulates in astroglial cells, disrupting myelin sheath formation. Even in very small concentrations, lead affects neural excitation and memory-related neurotransmitter activity [11].

### Toxicity of lead

Lead toxicity can cause acute or chronic health impacts. Though it can affect various organs in the body, it mainly leads to toxicity of the nervous, hematopoietic, cardiovascular, and other systems. In addition, recent studies have been reporting that lead is carcinogenic. While acute toxicity is uncommon, it occurs mainly due to occupational exposure, and in severe cases, it may cause death. Chronic toxicity occurs at relatively low levels, and there are quite frequent reports of the occurrence of negative health impacts, particularly in children, including the occurrence of neurobehavioral development abnormalities, at even lower blood lead levels.

#### Neurologic toxicity

The nervous system is the organ system most vulnerable to lead-induced toxicity [12]. While problems can occur in both the central and peripheral nervous systems, the peripheral nervous system is mainly affected in adults, whereas in children, the central nervous system is primarily affected [13]. When the impact on the central nervous system is severe, it manifests as encephalopathy. In relatively minor cases, symptoms such as headaches, irritability, muscular tremors, and loss of memory occur, whereas severe cases can result in delirium, convulsions, or coma [14]. Because their nervous system is still developing, children can be particularly vulnerable. Studies have reported that low levels of chronic exposure still have a negative effect on neurobehavioral development, such as lowered IQ and ADHD-like symptoms [2]. Resulting in demyelination, peripheral nervous system abnormalities frequently take the form of a peripheral neuropathy that invades the extensor motor nerves [15].

#### Hematologic toxicity

Lead causes anemia by dose-dependently blocking the activity of ferrochelatase, aminolevulinic acid synthetase (ALAS), and ALAD, three important enzymes related to the synthesis of heme. Among these, ALAD is the most markedly affected, and is therefore used as a clinical marker for lead poisoning. When ALAD is inhibited, aminolevulinic acid accumulates, and blood lead levels of 10 μg/dL and even lower begin to be detected [16]. Exposure to high levels of lead can lead to acute hemolytic anemia [17].

#### Cardiovascular toxicity

Lead raises the blood pressure, which increases the risk of death from cardiovascular disease. Lead exposure increases the frequency of high blood pressure, as well as cerebrovascular and cardiovascular disease [18]. An increase in blood lead levels has been shown to have a significant correlation with cardiovascular disease-related death [19]. Some findings have appeared to show a significant degree of correlation between blood lead levels and systolic and diastolic blood pressure [20]. There has been a great deal of research conducted on the relationship between low levels of lead exposure and blood pressure, and while there is still much that is up for debate, recent studies have found that low levels of lead exposure can cause high blood pressure [21]. The mechanism by which lead raises blood pressure is still not clearly understood. While some studies claim an association with chronic nephropathy [22], recent findings have suggested oxidative stress as the mechanism [21].

#### Other toxicity

Some studies have found lead to be a cause of reproductive toxicity in both men and women. In men, it causes a reduction in libido, infertility, as well as a reduction in sperm count and vigor, while in women there is an increase in the incidence of stillbirth and miscarriage [23]. Additionally, lead can cause a vitamin D deficiency by disrupting the conversion of vitamin D into its biologically active form, 1,25-dyhydroxyvitamin D [24].

#### Carcinogenicity

The International Agency for Research on Cancer (IARC) deems inorganic lead to be probably carcinogenic to humans (IARC Group 2A), based on sufficient evidence from animal testing and limited evidence with human subjects [1]. Lead smelters, plumbers, battery recycling smelters, pigment producers, and so on, are at risk to occupational exposure. Lead exposure is known to increase the risk of lung, stomach, and bladder cancer. Organic lead belongs to IARC Group 3, meaning that there is not sufficient evidence to evaluate whether or not it causes cancer in humans. However, a portion of organic lead can be metabolized as ionic lead, and in such instances, toxicity similar to that of inorganic lead can arise [1].

### Symptoms and signs of lead exposure

Symptoms of lead poisoning take a variety of forms based on the period of exposure and individual characteristics, and depending on the circumstances, non-specific or minor symptoms may appear, or there may even be cases with no noticeable symptoms whatsoever. In cases of chronic exposure, symptoms appear to become incrementally more severe as the weeks pass, whereas in cases of acute exposure, strong symptoms can suddenly appear. The degree or type of symptoms that patients complain of may differ depending on the form of the lead to which they are exposed. As organic lead has a higher lipid solubility compared with inorganic lead, its toxicity is stronger and its symptoms are mainly related to the central nervous system, such as insomnia, delirium, cognition problems, and tremors [25].

Symptoms manifest differently in adults than they do in children. In adults, major symptoms include headach, stomachach, memory loss, renal failure, sexual dysfunction, and reduced sensation in the limbs, and in the early period, non-specific symptoms may manifest such as depression, reduced appetite, intermittent stomachach, nausea, diarrhea, and constipation [25]. While symptoms generally manifest when blood lead levels exceed 40 μg/dL in adults and 60 μg/dL in children, symptoms may appear at different ranges depending on the individual’s characteristics [13]. Neuropsychological problems like delayed reaction time, reduced concentration, reduced neurotransmitter speed, and headaches first appear at blood lead levels of 25–60 μg/dL, anemia appears at blood lead levels of 50 μg/dL and over, colic in cases of 80 μg/dL and over, and intracranial hypertension and delirium begin to manifest when blood lead levels exceed 100 μg/dL, along with wrist drop, foot drop, and encephalopathy. In the case of children, neurological symptoms begin to manifest when blood lead levels exceed 70 μg/dL [26]. There are almost no instances of cases without symptoms once blood lead levels have exceeded 100 μg/dL, in either adults or children.

#### Acute exposure

During acute poisoning, neurological symptoms may occur such as pain, weakened muscle power, sensory abnormalities, and occasionally brain inflammation, along with digestive system-related symptoms such as stomachaches, nausea, vomiting, diarrhea, and constipation [27]. In instances where a large amount of lead has been ingested through the digestive system in a short period of time, the loss of a lot of water in the digestive system can cause shock, hemolytic action occurs, and symptoms such as anemia and hematuria may occur. In instances where the kidneys are damaged, the amount of urine decreases; individuals who showed symptoms of acute lead exposure can also show symptoms similar to those of chronic lead exposure [28].

#### Chronic exposure

In cases of chronic lead exposure, a variety of symptoms may manifest, mainly in the digestive system, nervous system, and neurological system. Neurological symptoms arise from a strong exposure over a relatively short time period. By contrast, digestive system symptoms generally arise from long-term exposure. In cases of chronic exposure over a long period of time, a variety of symptoms may manifest, including anemia, fatigue, reduced limb sensation, stomachache, nausea, vomiting, depression, and reduced concentration and memory [29]. Chronic lead poisoning may also manifest as a grey color in the skin or a blue line in the gums, known as the Burton line [30]. Aside from these symptoms, an increase in optic neuritis-induced scotoma and vision impairments may occur.

#### Lead exposure in pregnancy and children

Exposure of an embryo or fetus in utero to lead increases the possibility of premature birth and low birth weight [31]. As the body size of children is smaller than that of adults and lead is more quickly absorbed, they are more vulnerable to lead poisoning. Additionally, because babies crawl on the floor and suck on various objects, they are more readily exposed to lead. Common symptoms occurring in children include a loss of appetite, stomachache, vomiting, learning disabilities, behavioral problems, and anemia, while in abnormally high levels of lead exposure, symptoms of leukonychia striata are observed [32].

### Evaluation and diagnosis of lead exposure

Because the symptoms of lead poisoning do not appear instantly, even after having been exposed to lead, it is not easy to diagnose. Therefore, when lead poisoning is suspected, basic information should be determined through a medical interview that takes into consideration the symptoms reported by the patient, current medical history, and the surrounding environment or diet that could be associated with lead exposure. After a basic medical interview and examination, the lead concentrations are measured in the blood, hair, urine, and saliva. After confirming the presence of lead poisoning, the most important priority when observing progression is measuring the blood lead levels. Besides lead concentration levels, a number of other biomarkers are also measured during blood testing along with the lead concentration in the bone or soft tissue.

#### Lead concentration in the blood

Measuring blood lead levels is the most common method used for confirming the presence of lead. Currently, the blood lead level of a healthy adult who has not been exposed to a large amount of lead is 25 μg/dL or lower [33], and for children, the current threshold of 10 μg/dL or lower was revised to 5 μg/dL or lower [34]. The threshold for the occupational lead exposure group should not exceed 30 μg/dL during random blood testing, and NIOSH urged that the blood lead level for adults be lowered to under 10 μg/dL [35]. Looking at the measured blood lead levels of individuals exposed to lead chronically, levels of 30–80 μg/dL have been found in children living in old houses painted with lead-based paint, 77–104 μg/dL in pottery-glaze workers, 90–137 μg/dL in individuals who consumed contaminated herbal medicine, 109–139 μg/dL in indoor firing range instructors, and up to 330 μg/dL in individuals who had drunk juice from glazed pottery [36].

#### Lead concentration in bone

Lead is stored in two parts of the bone. Lead stored in the bone’s surface can readily pass into the blood, whereas lead stored in cortical bone is virtually immobilized. Studies have reported that 40–70 % of lead in the blood of adults originates from bones [37]. In the case of mothers, as the physiology of bone reabsorption differs, the percentage of lead in the blood that originates in the bones varies from 10–88 %, while 80 % or greater of the lead in the blood of fetuses and embryos is provided by the mother [38]. Lead exposure reduces bone density in adults, increasing the risk of osteoporosis [39].

Lead concentration in cortical bone can be measured through X-ray fluoroscopy (XRF). While lead in the blood does not reflect the degree to which lead has accumulated in the body at the time of the blood test [40], lead in cortical bone is meaningful because it can reflect the degree of long-term lead exposure and accumulation. Ethylenediaminetetraacetic acid (EDTA) mobilization testing and bone XRF are known to be the most sensitive techniques for determining the degree of lead accumulation [41].

#### Lead concentration in soft tissue

Lead also accumulates in soft tissue, the liver being the largest storage site at 33 %, and is stored in the kidneys, pancreas, ovaries, spleen, prostate, adrenal glands, brain, fatty tissue, testicles, heart, and skeletal muscle. It appears that lead concentrations in soft tissue remain similar throughout one’s lifetime [42].

#### Other biomarkers of lead exposure

Lead bonds with sulfhydryl proteins via divalent cations, thereby blocking protein structuration and enzyme action. The most representative example of this is the blocking of the action of the enzyme delta-aminolevulinic acid dehydratase (delta-ALAD; ALAD) during the process of heme synthesis. Impeding heme synthesis in this way is a major part of the mechanism of lead’s pathology, and if blood lead levels reach 20 μg/dL or higher, ALAD action is reduced by 50 % or more. However, because ALAD is not linearly related to the blood lead level, it is not utilized for diagnosis of lead poison [43].

Due to the decrease in ALAD, there is an increase in urinary aminolevulinic acid (ALA). As this phenomenon occurs at a blood lead level of 35 μg/dL or higher in adults and between 25 and 75 μg/dL in children, it cannot be utilized as a useful indicator of low-level lead poisoning. Blood ALA increases as blood ALAD decreases, and there appears to be a relation between the behavioral problems observed in lead poisoning and ALA’s reduction of gamma-Aminobutyric acid (GABA) secretion in the central nervous system [44]. In addition, the drop in heme synthesis leads to a drop in hemoglobin synthesis, which affects cellular respiration, and in cases of chronic lead exposure may cause fatigue and anemia. The level of ALAD expression in humans is influenced by the ALAD1 and ALAD2 genes, and studies have found that the ALAD2 gene raises blood lead levels and sensitivity to lead poisoning [45].

Ferrochelatase is an enzyme that inserts iron into protoporphyrin IX. This process is inhibited by lead, resulting in an increase in erythrocyte protoporphyrin (EP) and zinc protoporphyrin (ZPP). Due to such properties, EP and ZPP are used in the diagnosis of lead poisoning. However, the limitations of using EP and ZPP include the fact that blood lead levels must reach 30 μg/dL or greater in adults and 15 μg/dL in children for ZPP levels to increase, as well as the fact that a decline in heme synthesis is not limited to lead poisoning, and similar results can be found in cases such as porphyria, normal brain aging, hepatocirrhosis, and iron-deficiency [46].

Besides that, because lead lowers pyrimidine 5′-nucleotidase activity, blocking the maturation of red blood cells and ultimately causing anemia, basophilic stippling and hemolysis of immature red blood cells can be used as biomarkers. While basophilic stippling and hemocytolysis of immature red blood cells can occur from exposure to substances like benzene or arsenic, microcytic or normocytic-hypochromic anemia accompanied by basophilic stippling only occur in cases where the blood lead levels are 50 μg/dL in adults and 25–40 μg/dL or greater in children [47].

### Management of lead exposure: chelation

**Fig. 1:**
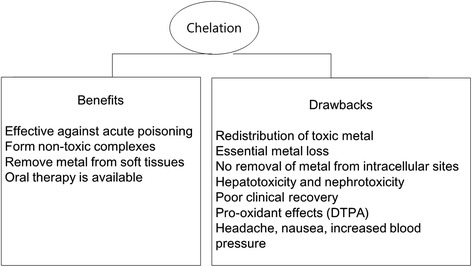
Benefits and drawbacks of chelation therapy

The majority of chelating agents bind to heavy metals in extracellular fluid and cannot cross the cellular membrane. Because they cause essential metal loss and may cause adverse drug effects such as hepatotoxicity or nephrotoxicity, while there are benefits to their use in cases of acute poisoning, they are not recommended for cases of chronic poisoning (Fig. 1) [48]. Due to the risk caused by the adverse side effects of the medicine and the redistribution of lead, chelation therapy is generally not recommended in cases where blood lead levels are below 45 μg/dL in adults [4]. Additionally, as rebounding commonly occurs after chelation therapy, blood lead levels should be measured before and after the treatment.

The drugs used as chelating agents for lead include dimercaptosuccinic acid (DMSA), dimercaptopropane sulfonate (DMPS), dimercaprol (British Anti-Lewisite, BAL), penicillamine, and CaNa_2_EDTA (Table 1).

**Table 1:** Overview of chelation drugs

Chemical name (common names, abbreviations)	Dose	Adverse effects	Elements chelated
2,3-bis(sulfanyl)butanedioic acid (Dimercaptosuccinic acid; DMSA; Succimer)	10 mg/kg (or 350 mg/m^2^) per 8 h for 5 days, then 10 mg/kg per 12 h for 14 days (a total of 19 days), PO^a^.	Gastrointestinal disturbances, mild increase in serum transaminase	Lead, arsenic, mercury, cadmium, silver, tin, copper
Sodium 2,3-bis(sulfanyl)propane-1-sulfonate (Dimercaptopropanesulfonate; DMPS; Dimaval)	Adult: 5 mg/kg per 6–8 h, PO, IM^b^, IV^c^, or SQ^d^.	Low back (kidney) pain, gastrointestinal disturbances, skin rash, fatigue, hypersensitivity reactions	Mercury, arsenic, lead, cadmium, tin, silver, copper, selenium, zinc, magnesium
	Children: 5-day course of 200 or 400 mg/m^2^/day		
2-[2-[bis(carboxymethyl)amino]ethyl-(carboxymethyl)amino]acetic acid (Ethylenediaminetetraacetic acid; EDTA; CaNa_2_EDTA)	1000–1500 mg/m^2^/day (1–2 g/24 h for a 70-kg adult) as an IV infusion for 5 days	Renal toxicity	Lead, cadmium, zinc
(2S)-2-amino-3-methyl-3-sulfanylbutanoic acid (3-Sulfanyl-D-valine; Penicillamine; D- Penicillamine)	10 mg/kg/day for 7 days with a possibility of a prolonged treatment during 2 to 3 weeks, PO.	Interstitial nephritis, hypersensitivity reactions, gastrointestinal disturbances, leukopenia and thrombocytopenia	Copper, arsenic, zinc, mercury, lead
2,3-bis(sulfanyl)propan-1-ol (Dimercaprol; British Anti-Lewisite; BAL)	50–75 mg/m^2^ per 4 h for 5 days, deep IM.	Allergy, gastrointestinal symptoms, tachycardia, fever, elevation of liver function tests	Arsenic, gold, mercury, lead (BAL in combination with CaNa_2_EDTA)

^a^ *PO* oral ingestion

^b^ *IM* intramuscular injection

^c^ *IV* intravenous injection

^d^ *SQ* subcutaneous injection

An analogue of dimercaprol, DMSA is a dithiol compound which contains two sulfhydryl (–SH) groups. DMSA has a large therapeutic window, and is the least toxic dithiol compound. While chelation therapy can result in a reduction in essential metals, DMSA has been found to not cause a reduction in essential metals such as zinc, iron, calcium, and magnesium [49]. DMSA is the most efficient and safe chelating agent for lead exposure, so the chelators being used the most are DMSA recently [50].

DMPS is another analogue of dimercaprol. Less effective than CaNa_2_EDTA or DMSA, DMPS is not commonly used in lead chelation therapy [48]. DMPS is mainly used in cases of arsenic or mercury poisoning.

The drawbacks of CaNa_2_EDTA are that it contributes to a greater loss in essential minerals compared to DMSA or DMPS and that it redistributes lead to the brain [50]. Generally, one gram of EDTA is mixed with 250 mL of a 5 % dextrose solution, gradually settling over one or two hours, and treatment is administered over a five day period. During the treatment period, the patient should be hydrated intravenously, and tests should be conducted daily on kidney function, electrolyte levels, and urine.

Penicillamine was the first chelating agent used in the treatment of lead poisoning. It reaches its greatest capacity one to four hours after oral administration. Food, antioxidants, and iron supplements can reduce absorption of penicillamine. In the past, the daily dose used was 500–1000 mg, but recent findings show that using a lower dosage had equivalent lead removal effects while reducing adverse side effects [51]. Due to the narrow therapeutic window and harsh side effects of dimercaprol (British Anti-Lewisite, BAL), it is rarely used these days.

In general, chelation therapy is recommended for children only in cases where blood lead levels are 45 μg/dL or greater. In cases where blood lead levels are 45–69 μg/dL, chelation therapy can be attempted with DMSA. McKay [52] tried chelation therapy on children with blood lead levels of 20–44 μg/dL. While the blood lead levels of the children who received chelation therapy decreased temporarily compared with the control group, when a follow-up study was conducted three years later, the blood lead levels of the therapy group had risen again, and no significant difference was observed in cognitive or behavioral function nor in blood lead levels between the therapy group and the control group.

While removing the source of lead exposure is most important for reducing the health impact of lead and similar harmful heavy metals, naturally occurring essential minerals (calcium, magnesium, selenium, etc.) and other related nutrient replacement therapies can be useful in decreasing the absorption of harmful heavy metals or promoting their excretion. New strategies in heavy metal chelation therapy include the use of structurally different chelating agents (combination therapy such as DMSA and EDTA) and the co-administration of antioxidants such as DMSA and taurine [50].

### Dietary management

Antioxidants are effective in alleviating and treating the oxidative stress-induced toxicity of lead. By interacting with generated reactive oxygen species (ROS), antioxidants prevent radical chain reactions. By chelating with lead ions, ROS generation is blocked, preventing and alleviating lead’s toxicity. While chelating agents can remove lead from the body, their effectiveness in treating lead’s neurological toxicity is unclear, and thus, they are not used in treatment for children with low blood lead concentrations [52]. Additionally, as chelating agents have a rebound effect, they cannot be used in instances where lead exposure cannot be suspended. However, antioxidants can be used in such instances where lead exposure cannot be suspended or in cases of low blood lead levels [5]. Antioxidants used in the alleviation of lead toxicity include vitamins, flavonoids, and herbs.

#### Vitamins

Vitamin B6 (pyridoxine) and B1 (thiamine) are effective in alleviating health problems caused by lead poisoning [5]. Vitamin B6 produces antioxidant effects by promoting GSH generation. In an animal study using rats as subjects, vitamin B1 was effective in alleviating lead-induced lipid peroxidation [53]. Lee et al. [54] found blood lead levels and homocysteine to have a proportional relationship in adults in the U.S., but as this showed different patterns depending on the vitamin B6, they suggested that an appropriate concentration of B6 in the body be maintained in order to block the effects of lead exposure.

Vitamin C (ascorbic acid) is the most widely studied antioxidant capable of removing free radicals. Unsurpassed in its ability to bind to and remove lead, vitamin C is highly effective at alleviating lead toxicity [55]. Through its antioxidant activity, vitamin C improves the lead-induced impairments in synaptic plasticity. The combined administration of vitamin C with silymarin can alleviate lead-induced hepatotoxicity [56]. In a study by Tandon et al. [57], lead-poisoned patients were administered 250 mg of vitamin C two times daily for a month, which resulted in a reduction in blood lead levels and an increase in blood ALAD activity. One study found that administering 250–500 mg of vitamin C daily to children was effective in removing free radicals and in treating lead-induced health problems [58].

A fat-soluble vitamin, vitamin E (α-tocopherol) exhibits powerful antioxidant effects. With its neuro-protective effect and antioxidant effect, vitamin E improves cognitive impairment caused by aging [59]. Sajitha et al. [60] reported an improvement in lipid levels and the alleviation of lipid peroxidation-induced liver, heart, and kidney impairment in rats that had been administered vitamin E. As vitamin E improves lead-induced memory impairment, it has been recommended as good for preventing lead-induced health problems with appropriate dosing [61].

#### Flavonoids

Natural polyphenolic compounds, flavonoids are abundant in fruits and vegetables. Like other antioxidants, flavonoids prevent oxidative stress by binding with heavy metal ions and preventing free radical chain reactions. Quercetin is abundant in fruit, vegetables, and tea. It has a stronger antioxidant effect than vitamin C or E. Not merely possessing antioxidant effects, quercetin also has anti-inflammatory properties, and findings show that it is effective for diseases such as ischemic heart disease, atherosclerosis, hepatocirrhosis, and nephritis [62, 63]. Quercetin is effective in alleviating lead-induced liver, kidney, and brain damage [64, 65]. In particular, as quercetin crosses the blood-brain barrier, it can alleviate toxicity in the brain by being able to bind to some of the lead accumulated in the hippocampus [66]. Though the optimal dosage has not been settled, the oral ingestion of quercetin is known to cause no particular side effects [64]. Therefore, with almost no adverse side effects, being extremely effective at removing lead and treating lead-induced toxicity, quercetin is a medicine worth recommending when lead exposure is suspected.

An antioxidant that can be used in the treatment and prevention of oxidative stress-induced disease, α-lipoic acid is found in foods like carrots, beets, and potatoes. While lipoid acid blocks oxidative stress, as it does not chelate to heavy metals, it is often used in combination with chelating agents, DMSA (2,3-dimercaptosuccinic acid) in particular. According to previous studies, lipoic acid used in combination with DMSA showed excellent effectiveness in alleviating lead-induced oxidative stress [67], as well as having similar effects in other heavy metals (arsenic) besides lead [68]. Pande and Flora [69] reported that a combination of lipoic acid and thiol chelator was able to alleviate lead toxicity, and in particular, it reduced oxidative stress and lead levels in the brain.

#### Herbs

Inexpensive and with few adverse side effects, herbs are being used clinically with effect. However, as the treatment period is very long, their use is limited to prevention rather than treatment of symptoms of lead exposure.

Garlic is mostly used for cooking, but due to its antioxidant effect, it is also widely used as an antioxidant. Garlic is effective in preventing lead-induced impairment of the liver or reproductive organs [70]. In an investigation into the herbal intake and blood lead levels in U.S. adults, Buettner et al. [71] found that, unlike with other herbs, the blood lead level of adults who had ingested garlic did not increase, and stated that this finding implies the efficacy of garlic in controlling blood lead levels. In a study where garlic and D-penicillamine were administered over a period of four weeks to two groups of patients with chronic lead poisoning, Kianoush et al. [72] reported that, while blood lead levels were reduced in both groups, the levels were lower in the garlic-administered group.

A substance from the spice derived from the rhizomes of a plant known as curcuma longa, curcumin is a polyphenol compound. Sukla et al. [73] found via animal studies that curcumin is effective in preventing lead-induced neurotoxicity. Since those findings, many studies have been carried out looking at curcumin and lead toxicity.

## Conclusions

Many studies have been conducted on the occupational exposure and health impact of lead, and the various efforts to prevent health problems caused by lead resulted in the significant decrease in the number of cases of occupational lead poisoning. As interest has grown in lead’s carcinogenicity and the health problems of environmental lead exposure and exposure to low concentrations of lead, efforts are underway to reduce the concentrations of lead in the environment. Recently, studies have been conducted on health problems caused by low-level lead exposure, and reports on long-term low-level lead exposure and a variety of health problems are being continuously published. In this study we examined the toxicity and health impact of lead, and reviewed recent literature on the observation and treatment of lead exposure. We expect that this study can be usefully applied to the observation and management of environmental lead exposure.
